# Pathology

**Published:** 1870-07

**Authors:** W. H. Atkinson


DENTAL REGISTER.

Vol. XXIV.] JULY, 1870. [No. 7.

### Original Communications.

### PATHOLOGY.

BY PROF. W. H. ATKINSON.

Pathology being the essential condition of living bodies that lays the foundation for, and prescribes the laws of remedy or cure, it behooves the men who engage in the administration of preventives or remedies, to acquaint themselves with all and singular the inceptions, developments, and culminations of the conditions called pathological. To do this in any useful degree we must locate the process called function; which involves an understanding of the origin, growth, nourishment, and decadence of the bodies that elaborate function. The object of function is to satisfy or harmonize tenant with tenement; type with structure; ghost with habitat. And hence, legitimately, physiology when pure in expression, pathology when in minus or mixed manifestation. In other words, pathology is a compromise between physiology and death of the body, or bodies, under its dominion.

It is now necessary for us to define what is meant by the term body. In the first and most general sense it signifies the human system, or physical body, the flesh, the form. Secondarily, any organ of the human system. Thirdly, any tissue; and Fourthly, the elements of tissues that characterize and distinguish them, denominated cells.

Cells are formed out of, and nourished by, the appropriation of amorphous elemental substance variously denominated blastema, cytoblastema, plasma, plasm, mucus, pabulum, neural mass, juices of the flesh, etc., etc.

In this sea of unpronounced body, or possibility of all forms of body, a process of differentiation takes place by which each variety of primate of tissue and organ is produced.

The first apparent change is formation of minute bodies called granules, or "granulous matter." The next is the accumulation of these in small groups, that obstruct the light, and appear dark or black, according to their power of absorbing or deffracting or reflecting the light. These are called nuclei, or centers to which blastema and granulous matter are attracted on all sides, which is now denominated a nucleated cell. After this a still farther segregation and condensation on the outer border of this body, of this same germinal matter, takes place, producing the so called cell-wall; and now we may say the cell is formed. In many cells a yet darker little body makes its appearance within the nucleus, which has been named nucleolus, or little nucleus. These then are the essential elemental bodies that are concerned in the functioning of nutrient processes. Beginning at the lowest and least, and rising to the highest and greatest bodies that are concerned in the elaboration of function in the human system, they may be annotated thus: 1. Blastema; 2. Granule; 3. Nucleus; 4. Cell; 5. Tissue. 6. Organ; 7. System; 8. Mind. Each depending on its immediate predecessor in the scale, and all having an outer coat, or atmosphere of the first, (blastema), except the 7th, which is immersed in, or surrounded by the atmosphere proper of the earth, from which to draw supplies, and into which to cast off excessive quantity or offensive quality of pabulum and the debris resultant upon functional activity.

This is the machinery that elaborates functional process in the bodies of men. And as man is a proper epitome of the universe we may confidently look for examples of planetary processes in diffierential display in the system as a whole, and in the parts of which it is constituted.

Naturalists have divided planetary existences into three kingdoms; namely: Mineral, Vegetable and Animal. Without fully endorsing this nomination as satisfactory, it will serve our present purpose in preference to demolishing it and erecting a better on the ruins. The mode of formation of primary bodies is simple accretion of the elemental particles of which they are composed. The completest expression of accretion is nothing less than crystallization, and this is the mode of formation of the mineral kingdom. All that can be invoked as the nutrition or maintainance of the integrity of the crystal is the continuity, indefinitely, of that tendency, or togetherness that first induced the elemental particles to come together in a solid condition. Separation of the atoms can only be effected by solutions, and the gyrations by heat, reducing the crystal to liquidity, or gaseous conditions. Recrystallizations are resurrections of crystals, but not resuscitation thereof; that is to say a reproduction of similar bodies from the solution or supernatant liquid, but not a building in of lost portions of the atoms of the identical bodies that had been dissolved.

Solution of crystals is not the best term to indicate the dissolution or death of a crystal. Solution being the first degree of dissolution of a solid, or first refusal on the side of the particles to part company by farther and farther diffusion into gases, ethers, etc.; each marking a stage of opposition to the destructive force.

This tendency to maintain the integrity of crystals is a triple intercurrental movement of Chemism, Magnetism, and Electrism, or a balancing of the tensions of the center substance and surface of the nucleus, or oldest part of the crystal, whereby its solidity is maintained. It thus becomes a point of attraction to the particles diffused in the mother lye so that new layers of solid particles may be regularly laid down upon all sides which maintain the specific character of the crystal, and enables us to recognize it by its facets and other indications. The class of bodies next in the ascending order of endowment of this force, or tendency to associate among particles is displayed in the the vegetable cell; in which the order of accretion is divided by a half reversal and a half repetition of the former process; that is to say by layers on the inner surface and the outer surface of the cell; a true endogenous and exogenous growth. When a vegetable cell is once completed it assumes a state of solidity, increasing until it assimilates so nearly the status of a crystal as to almost classify it among minerals.

In the animal all the life entities that in their united capacity endow it as such, are so diverse and humerous as to resist the tendency to solidity to such a degree as to expel it by removing the worn out and weary particles by solution and dispersion, to prevent their retrogression to the vegetable or mineral status of endowment. This is displayed in what has been called alternations of generation of cells, out of which arises the mobility and renewals of the tissues; and is creating and uncreating these elements of tissue, organ, and body. In the mineral there is but a single tendency of togetherness, and that is perpetual. In the vegetable there is a to and fro tendency during the formation of the tissues, but as soon as they are complete they tend toward the state of quiescence of the mineral kingdom; while the animal cell attracts, examines, and accepts or rejects the portions of pabulum brought within its sphere. If accepted it is put in place to perform the needed work, to sustain function, and kept there so long as fit for use. The manner in which it is disposed of depends upon quantity and quality of the supply; as a rule it is rejected so soon as it falls to the level of the vegetable or mineral measure of endowment. Rejection is effected in the normal way by expulsion from the outlets of the body without constitutional disturbance, but if this process is resisted the effete or excessive portions of pabulum is allowed to accumulate in place, until it arouses the expulsive effort; or one that shuts them up in cysts, (the origin of tumors).

The range and diversity of life endowment in the various departments of the human body is so great as to render it impossible to supply them all from a common fountain with pure pabulum, commensurate with their needs. All foods to be amenable to appropriation must be reduced to a tension of togetherness and apartness, acquiesing with that of the tissues to which they must be assimilated to constitute normal nourishment. We have already seen that there are modes of aggregation of the constituents of this body, analagous to those holding dominion in the mineral, vegetable, and animal kingdoms. Also that there are departments which are held together for use by these identical laws; so that to be able to comprehend them we must understand crystallization, solutions, (or liquidity) gasidity and etherealization, as they occur in the making and unmaking of the elements of the bodies in which functional metamorphoses take place. Going from the highest and greatest (aggregate) to the lowest and least (individual) constituent of our minds and bodies, we involve: 1st. Spiritual process. 2d. Mental P. 3d. Systemic P. 4th. Organic P. 5th. Tissual P. 6th. Cell processes; and 7th. Blastemal processes.

Harmonial movement of all these produces health, inharmonial disease, or pathology. When we contemplate the immensity of range and diversity of influx that must help or hurt, or hinder these processes, is it any marvel that disease, of greater or less virulence, holds such general dominion of the field of spiritual, mental, and physical functions among men?

It was intimated that each body, and element of body, has an atmosphere from which it inspires that which serves it as food, or acts as poison. This is strictly so respecting both the seen and unseen departments of being, spiritual, mental, systemic, organic, etc., etc., down to the unseen atoms in blastema, whose aggregation constitutes our granule or molecule out of which cell and tissue are constituted; no less than the unseen spirit and soul which together, as type, issues the mandate by which amorphous matter becomes blastema, granule, etc.; up to the complete human body, so wonderfully adapted to locate and elaborate the functions of health and disease, in accordance with the purity or impurity of that which we denominate food, (pabulum proper.) To be more specific and within the range of the shortest sight, air is the pabulum of lung, liquid of vessel, semi-solid of cell and solid of crystal. Thus all possibility of bodily food is resolvable into solids, semi-solids, or colloids, liquids and gases. The nutrition of the body and its parts is nothing less than this veritable metamorphosis in alternations of construction and destruction, and reconstruction of the tissues that constitute them. Digestion is the proper placing of the constituents of the cells and tissues. Work is the maintaining of these elements in place, or removing them as required, (in renewals); to secure the harmony of the system these together with the complications of, and reciprocations with the parts and the whole, or oppositions or disobedience to the laws and processes involved, constitute *function* in general, and in particular in physiological and pathological display.

The state of the tissue to be nourished must be taken into the calculation, if we would comprehend abnormities in nutrient actions. The full body refuses even the daintiest foods, while the empty body takes without much hesitation, that with which it would be disgusted in moderate state of need. Just to what degree it possesses the power to render innocent and nutrient that which would be poisonous and deteriorating depends upon the local and general conditions and degree of activity of the parts and of the whole. Even very virulent poisons are eliminated from the body under violent and long sustained exercise, which prove fatal if the system be kept at rest.

Just what degree of intelligence resides in the granules which disposes them to answer each others attraction when coming together to form cells, is not so readily measured, but that they know just what degree of nearness renders them efficient in the production of the cells, and these of tissues and these of organs, and finally these of the entire system is too apparent to admit of a doubt. When these elements are concordant they come together and form the societies which constitute, first, the parts and then the cell, and they dwell together in harmony for a time, until each shall be so interpenetrated with the quantum of force and form resident in other as to render them so nearly alike as to make them repellent to each other.

This is the diastole of the cell, and becomes the opportunity to surrounding pabulum to inflow and stimulate the systole of the cell, by which the exhausted granulous matter, or debris of worn or weakened molecules, is thrown into the sluiceways provided in the intercellular spaces for this very end. This completes the specific function of the cell for itself. And if no further process be added, the role of which we have just spoken would continue as long as the supply of pubulum was continued. That which may be called the ageing of cells has already been referred to in speaking of vegetable growth; and in accounting for the alternations of generations of cells in animal forms of being. The intercurrental lines of tension of force and form, can not equally charge the molecules at the center, surface, and between, so as to make them all move simultaneously and regularly apart, and admit of the influx of fresh granular mass, to supply the parts with newly constructed nucleoli, nuclei, parenchyma, and cell-wall. In consequence of this the ageing of the elements will not affect all alike, so that some will be more used up, worn or weary, than others.

These effete members of the little community either refuse to move into the outer territories, and thus interfere by statical obstructions with the local function; or move with accelerated celerity into the sluice-ways. And thus we have admitted an irregularity as to time of ripening or waning of the different parts of the cell, so that a portion is nearly destitute of diastolic and systolic movement, and other portions so very active as to effect nutrient change so rapidly as to pass many alternations and renewals, while the former has fallen in nutrient activity, almost to the merely vegetable mode of sustaining life.

Thus we see that portions continue to act specifically for the individual life of the cell, while a higher function, namely, that of tissue, is initiated by the slowing of the peripheral process in nutrient movement, which enables them to join like portions of other cells, and thus produce filaments of tissue, that are sustained by percolation, imbibition, and assimilation of pabulum. Percolation among the filaments, imbibition by cell wall, parenchyma, nucleus and contents; assimilation in fibre, wall, nucleolus, nucleus, parenchyma, and granular mass. This statement, when understood, and established in the mind, will explain all the phenomena and processes of growth and nutrition by applying it to the cells, tissues, organs, and the entire body; and prepare us to apprehend the helpful or hurtful action of dynamic, mental, chemical, magnetic, electric and mechanical influx, with all their degrees and modifications. Fullness and deficiency being the extremes of endowment are the examples of satisfaction and dissatisfaction.

As the attainment of satisfaction is the object of all work affectional, mental, and physical, it will readily be perceived that the simpler the need the easier it is to satisfy it; and the more complicated and diverse the necessity the greater and more difficult will be the labor to meet the demand.

In the light of these statements it will be seen that the mineralistic, vegetableistic, animalistic, humanistic, and dynamical departments of our being are attained and maintained with a facility commensurate with their simplicity and complications.

Each tissue must be formed from and fed by the material of which it consists, and held in relation to the next in order by a compromise between the degrees of endowment of each, else each must stand alone as independent system, drawing its supplies directly from the air or water, in which it lives. Were this not so we might have enamel in contact with pulp, epithelium upon muscle, without intervention of basement, membrane, and nerve pulp, or neural mass without cylinder or axis; all harmoniously agreeing to have nothing to do in common, and hence not in possession of any bond of union that the slightest jar would not knock into interminable confusion.

Instead of this we have a plan by which each department is joined to the others by an accommodating material, capable of affiliation with its diverse neighbors on either hand. This connective tissue may be very soft or very hard, and possessed of a tenacity to life quite remarkable; ready to entertain mineral modes on the one hand and animal on the other, of nutrient activity, while it vegetates in its interior on its own account, which preserves its peculiar character and power.

This plan has ordained that there shall be a fitness of every part to perform the use to which it is destined.

In the enamel we have the synonym of the mineral kingdom, in the dentine and bones a sort of compromise between mineral and vegetable modes of life; in the cellular tissues one between this last and the animal mode; and in the soft tissues, in general, the type of animal nutrient activity. The teeth are suited to their office of cutting, tearing, and grinding: the bones to supporting and protecting the soft parts, and these to perform the many varieties of solving and rendering miscible the bodies which the teeth have broken, that the elements therein combined may subserve the uses of the economy in the secretions, nourishment, and excretions requisite to sustain the composite acts of mental and bodily function.

The origin of function, like the origin of body, comes from the ocean of the unseen, and whether it is formed by another, or forms itself, need not engage present attention. But that a body must be, before it can act in a specific manner is as evident as that mind must exist and be in action to cognize its own labors. In a word, wherever function is present that which actuates function is already premised, or posited.

Obedience to the mandate of law or authority can only be secured in its purity by two methods, namely: spontaniety, or voluntary consent.

The highest obedience is the result of these conjointly, and vividly responding instantly to the impulse of the command.

That which we call original thinking holds a very close similitude to the origin of the elements of physical body, in the transparent ocean of chaotic elemental mass in which it first appears to sight and sense; namely, granule in blastema.

When the mental philosopher is interrogated for the origin of his discoveries, his best answer usually is: "It came into my mind, and the more I revolved it over the more apparent became the certitude of the propositions that were presented to me!"

Let us ask whence came the conceptions that prove to be legitimate transcripts of the departments of mind and matter to which they relate?

That they came from the common source of life of all individual existences may readily be proven, by a simple statement. That is, that a persistent yielding to distressing mental tension will result in debility, disease, and death of even the most robust organism. Happily for us we are not able to accomplish this dangerous feat without more persistence than it is given the many to exert. But let us improve the lesson it contains for our use in this most difficult of problems to solve, viz: the source of life and motion.

It seems to me that conceptions are introduced into our consciousness from the spirit world, by impregnating the nerve aura within us which constitutes our sentient centers, corresponding to the sensitive surface or film on the photographer's plate, where they, like it, are impressed with the image, shadow, or similitude of that which is evolved from or stamped upon them by the influx and the resistence (or resilience) thereto, resident in this most indifferenced and spiritual territory of our personality; in other words, by receiving and absorbing the influx, just as the sensitive film receives and stores up the sun-presence which depicts the linaments of the objects within the focus of the light pencil, so as to retain them for observation and review.

Now the recalling, reviewing, repeating, or recollection of the emotional movements, or mental labor, produced as stated, constitutes memory or recognition of the cognitions that precede all voluntary mental processes, and which are therefore purely spontaneous.

To pursue the simile; the specific affinities resident in the light and the quick stuff of the photographer's plate, are instantly satisfied, and were the mere satisfaction of these the required result we might proceed no further with the process; but that we may look upon the image it must be developed, as the artists say. That is, the unchanged quick stuff must be washed away, or a farther deposit must be made to bring out the picture, that it may be of use.

In like manner, if the satisfaction of influx and resistence "per se," were the object of this spontaneous mentality, a continued series, without repetitions, would supply the demand, and the process could not extend in more than a single direction, and then the universe of mind and matter would remain forever in an atomic state of nebulous existence the atoms running eternally in parallel lines preventing contact and blendings, both of which must be secured to people space with suns and systems that mutually hold each other in balance and check, to produce the cosmical idea of a universe in which every part is the exact counterpart of the rest; and alternately most important and least important in the functioning of the harmony of the spheres of which the universe is constituted.

The very idea of function, be it that which has been called mental or physical, involves change, metamorphosis. The fact is we can not separate these two elements of metamorphosis without annihilating the process. For all change must have a cause, a motive, and that it shall be regular and constant, a plan or idea must precede, produce, and preside in each and every change, infinitesimal or infinite.

The origin of *plan* in the case of the photographer is any object that arrests the pencil of light sufficiently strong to cast a shadow, or be visible, as we say; (capable of sensuous cognition.) The *plan* of suns and planets, and systems of planets, with their floræ and faunæ, and systems and cycles, of literatures describing, and attempting to account for all these (which are shed along the pathway of careering inquiry like forest leaves in autumn) is one, and capable of being read by the humility that sees the largest foreshadowed in the least, and the least repeated in the greatest metamorphosis in matter and mind, as but aspectings of the universe.

When metamorphosis shall have been fully comprehended it will lay open to view the origin and progress of variety, species, genera, etc., etc.; from planet, sun, and system, to most multitudinous inhabitants of planet, mineral, vegetable, animal, and man, as parasite and host, from granule, spore, and ovum, through every stage of alternation of generations, of parts to the whole, or completed stage of individual body, mineral, plant, or animal, up to man, who so grandiloquently denies his oval, deutoval, tritoval, and other metamorphosis of the histolysis of the parts that constitute him.

The great difficulty that stands in the way of a real progress has been so long blindly accepted, or adopted, that it is well nigh impossible to force it to give place to the truth.

This stumbling-block is the old assumption that "in time past God communicated directly to His creatures the truths of science, and religion," and then closed the avenues of communication, and left us, ever after, the only alternative of following the cabalistic jargon of the puerile estate, or mope forever in painful uncertainty.

The whole world of science has groaned, being burthened with this intolerable load, (with few exceptions), until now desiring to be free from the inherited disability of formula and uncancelled primary propositions, whereby alone we attain the demonstrations sought; only because we dare not follow, implicitly, the spontaneous lead that would open our vision to perceive that science is not real but phenomenal; not living but dead; not knowledge, simply rule; order, not truth, merely fact; not being, only appearance.

The old philosophy was forced to assume that a communication was necessary to assert with verity any formula; but it forgot to take into the account, to state, that there must be some "illumination" of the understanding to comprehend even that which the formula, or statement, contained, or signified. It is this illumination that confers the ability which enables us to give assent to or dissent from doctrines, or formula; in a word, to perceive what is meant. Until we perceive that which is called discovery to be communication, we shall be unable to get above the old philosophy; which regards this earth-life, or state as a finality, or result of creative energy and purpose; not as it really is, only a process of involution and evolution of body, mind, and conscience, for the enlargement of the individual, to fellowship with the Divine; and not a creation of mere creatures to adore the Creator! Science, as such, will never set us free, but religion has this very purpose and use.

All that is now proved aphorism was once vague speculation in the minds of those to whom it was given, or those who took it upon themselves to reduce it to form, that it might be the more readily communicated to others. As all the minor propositions in science have to die by cancellations into the higher, and these in their turn pass the same ordeal, we must not be discouraged to find that our highest and best attainments are subject to a like process of death, to become the basis of still more extended views and understanding.

The vicarious performances of function in plants and animals, in which parts exchange, assume, or repeat offices not normal to them, is too well known to naturalists to require more than mention to entitle them to be regarded as prophesies of coming types above in regeneration, and below in degeneration of the activities as they occur in nutrition and denutrition of the parts.

This meagre outlook upon the field of function will help us to see that we are under the influence of conscience, intellect, and appetite, to an extent little heeded by those who make haste to reap where they have not sown the seeds of philosophy with the assiduity and patience that can alone enable them to give themselves implicitly to the guidance of spontaneous influx, which always raises to higher, and higher states, when unchecked, and thus establishes a physiological state.

Our moral, mental, and physical life then, is not an inheritance or a possession, limited by the type of parentage in endowment, and therefore a fixed or calculable quantity and quality; but an ever advancing, or ever receding status, as we kindly follow first impulses, or stop to calculate consequences and thus take our destiny into our own hands, by substituting our ignorance for the luminous intelligence of the angels that proffer us the abundance of life. And thus, by having our own way, we fall into divers difficulties and disease, instead of spontaneously accepting life and being to our fullest capacity.

